# Umbilical outpouchings in pigs – an international survey on fitness for transport, welfare and management

**DOI:** 10.1186/s40813-024-00411-8

**Published:** 2025-01-09

**Authors:** Marie-Louise Hansen, Inge Larsen, Tina Birk Jensen, Charlotte Sonne Kristensen, Ken Steen Pedersen

**Affiliations:** 1 Department of Veterinary and Animal Sciences, Faculty of Health and Medical Sciences, University of Copenhagen, Grønnegårdsvej 2, Frederiksberg C, 1870 Denmark; 2 SEGES Innovation, Agro Food Park 15, Aarhus, 8200 Denmark

**Keywords:** Umbilical outpouchings, pigs, prevention, fitness for transport, welfare

## Abstract

**Background:**

Umbilical outpouchings (UOs) in pigs are a multifactorial disease and little is known about effective prevention strategies and risk factors for UO development. UOs are common in Danish pigs and legislation complicates and increases the cost of keeping and raising pigs with UO. Recommendations for preventive measures exist but the scientific evidence behind the recommendations is often lacking. This study´s purpose was to investigate veterinarians’ perspectives on UO pigs´ fitness for transport, the welfare of UO pigs, the significance of UO, risk factors for the development of UO, and the management of pigs with UO. This study´s purpose was to investigate veterinarians’ perspectives on UO pigs´ fitness for transport, the welfare of UO pigs, the significance of UO, risk factors for the development of UO, and the management of pigs with UO.

**Results:**

The survey received 93 complete responses from veterinarians working within porcine health management. Because of the large proportion of replies from Danish veterinarians, all reporting of results was divided among Danish and non-Danish veterinarians. There were no statistically significant differences between the two groups in the assessment of fitness for transport. Both groups mainly agreed to a series of statements regarding the significance of UO and risk factors for UO development. The management of UO was also similar across the groups except for the use of neonatal antibiotics which was used considerably more in Danish herds.

**Conclusions:**

Umbilical outpouchings seem to be perceived as a challenge across pork production; affecting the welfare of the individual pig as well as the management of the entire production. There were no significant differences between Danish and non-Danish veterinarians’ assessments of fitness for transport, and almost all the veterinarians agreed that some UO pigs might need special attention and care. Most would also recommend preventive measures. Despite most respondents in this survey working under the laws of the European Union, some were unaware of legislation regarding UO pigs.

## Background

Umbilical outpouchings (UOs) in pigs are a multifactorial disease and little is known about effective prevention strategies and risk factors for UO development. The extent of the problem is poorly illuminated, but research suggests that UO affects approximately a million Danish pigs yearly [1]; pigs that require special attention and care and might have reduced welfare [2]. At the European level Council Regulation no 1/2005 [3] and Council Directive 2008/120 [4] form the basic rules for transport of animals and minimum standards for protection of pigs. These are enforced and tightened in the Danish legislation where pigs with large UOs must be housed in hospital pens and can only be transported provided specific conditions (directly to slaughter, more space, soft bedding, and a veterinary certificate) are met [2, 5–7], and if the UO affect general conditions, reduces growth or impedes movement, the pig must be euthanised immediately [2]. UO pigs therefore contribute to a higher mortality and a poorer economy in pork production, which makes it important to identify measures that can reduce or eliminate the problem. Recommendations exist but most are not based on peer-reviewed studies [8]. The standard regimen with metaphylactic antibiotics for new-born piglets [1] is under scrutiny because of the growing concerns of antibiotic consumption and a focus on more sustainable pig production. One of the main purposes of neonatal antibiotics is the prevention of UO hypothesized to be caused by an umbilical infection. However, the effect of neonatal antibiotics on preventing UO has been the subject of mixed conclusions in earlier research [9–13] and secondly, treating all animals with antibiotics as standard is not considered prudent use of antibiotics.

The purpose of this study was to investigate veterinarians, working in different countries, perspectives on UO pigs´ fitness for transport, the welfare of UO pigs, the significance of UO, risk factors for the development of UO, and the management of pigs with UO.

## Results

The survey was distributed in the summer of 2021 and received 93 complete responses from veterinarians working within porcine health management. Most of the veterinarians were full or part-time practising veterinarians (*n* = 64), whereas the remaining veterinarians were distributed among scientists (private/ university, *n* = 15), the medical industry (*n* = 9), PhD students (*n* = 2), and others (*n* = 3). Most of the veterinarians were from Denmark (*n* = 52), but replies were also received from Australia (*n* = 1), Belgium (*n* = 1), Canada (*n* = 2), Finland (*n* = 4), France (*n* = 5), Germany (*n* = 3), Greece (*n* = 1), Ireland (*n* = 4), Italy (*n* = 1), Netherlands (*n* = 2), Norway (*n* = 1), Republic of Moldova (*n* = 1), Spain (*n* = 1), Sweden (*n* = 2), Switzerland (*n* = 2), United Kingdom (*n* = 6), USA (*n* = 1) and unknown (*n* = 3).

Due to the large proportion of Danish veterinarians, all reporting of results is divided between Danish veterinarians and non-Danish veterinarians.

### Fitness for transport

**Fig. 1:**
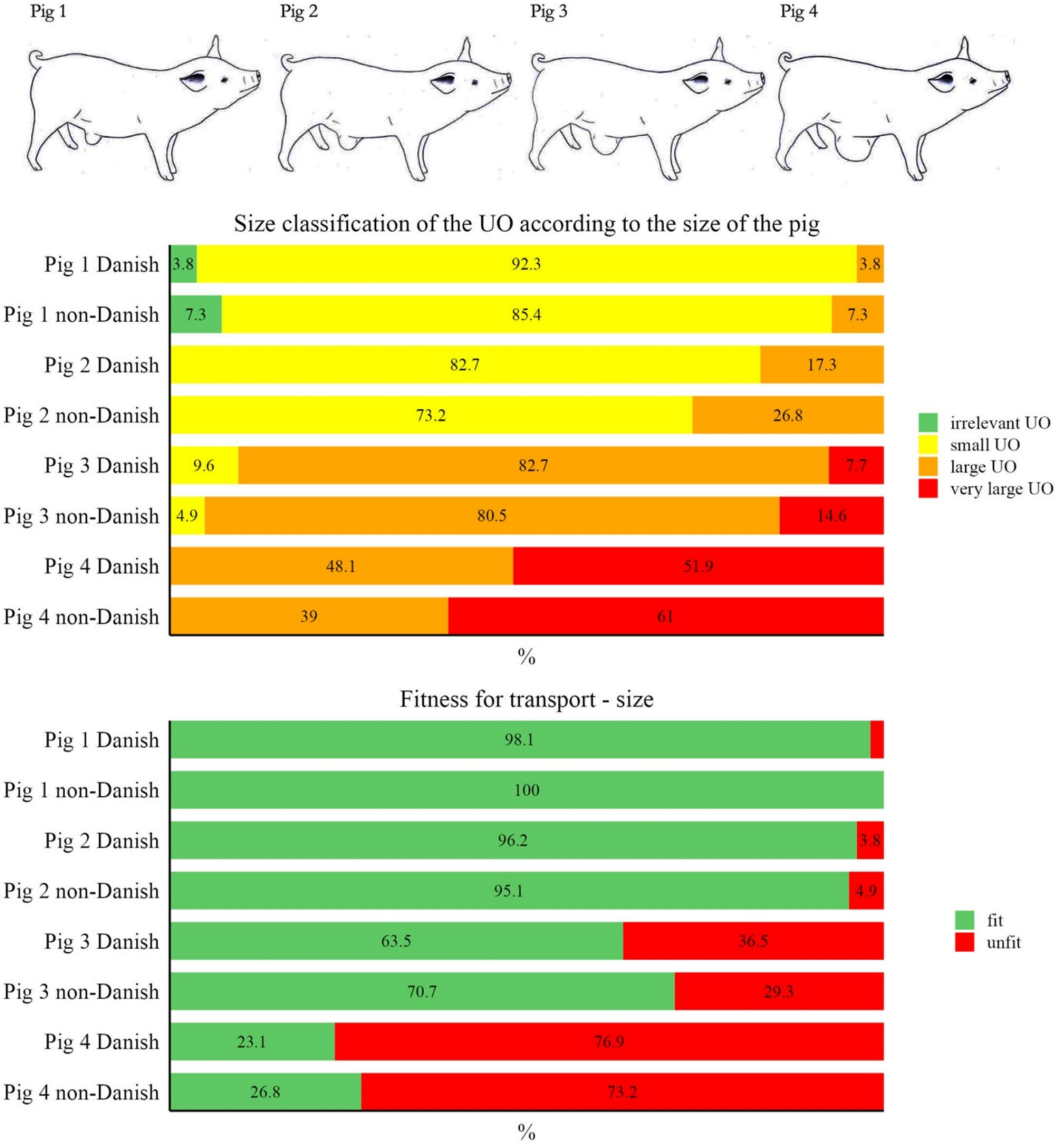
Results from the assessment of umbilical outpouching (UO) size and fitness for transport based on four images of pigs, evaluated by 52 Danish and 41 non-Danish veterinarians

The veterinarians were asked to classify the size of the UOs in four pictures of pigs and to indicate whether they considered the pigs fit for transport. Figure 1 shows the answer to the classification of size as well as the fitness for transport assessment.

**Fig. 2:**
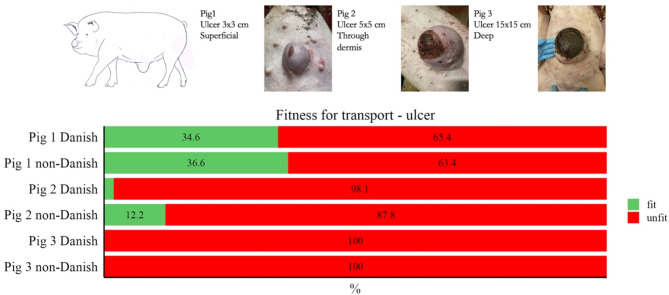
Results from the assessment of fitness for transport based on three images of pigs with ulcers, evaluated by 52 Danish and 41 non-Danish veterinarians

The veterinarians were asked to give their opinion on the transport fitness of three pigs with ulcers of different sizes, shown in images. Their answers are shown in Fig. 2.

The veterinarians were asked to rank three statements in order of relevance when assessing fitness for transport. Their answers are shown in Table 1. The veterinarians could also add factors they considered important. Ulcers and the welfare of the pigs were considered important factors as well.

**Table 1:** Results from 52 Danish and 41 non-Danish veterinarians ranking three statements relevant when assessing fitness for transport

		Importance % (*n*)			
Statement	Group	Most	Medium	Least	Total
Size of UO relative to size of pig	Danish	51.9 (27)	40.4 (21)	7.7 (4)	100 (52)
	non-Danish	58.5 (24)	21.9 (9)	19.5 (8)	100 (41)
How close the UO is to the ground	Danish	34.6 (18)	15.4 (8)	50.0 (26)	100 (52)
	non-Danish	29.3 (12)	19.5 (8)	51.2 (21)	100 (41)
The UO affects the ability to walk normally	Danish	13.5 (7)	44.2 (23)	42.3 (22)	100 (52)
	non-Danish	12.2 (5)	58.5 (24)	29.3 (12)	100 (41)

All respondents were asked how many cm between the lowest point of the UO and the floor they would find acceptable during transport, and whether UOs above a certain size should always be considered problematic; 21 (40.4%) of the Danish veterinarians and 24 (58.5%) of the non-Danish veterinarians agreed that a certain size should always be considered problematic. Table 2 shows the range, median and mean values in cm for acceptable distances to the floor during transport as well as the range, median and mean for problematic size of UOs.

**Table 2:** results from suggested distance UO to floor and size considerations for problematic UO

		cm			
Statement	Group	Range	Median	Mean	% (n)
Distance UO – floor *weaner*	Danish	3–20	10	10.1	92.3 (48)
	non-Danish	3–20	10	11.2	90.2 (37)
Distance UO – floor *finisher*	Danish	5–25	10	11.3	92.3 (48)
	non-Danish	5–40	20	18.2	95 (39)
Certain size	Danish	5–25	10	11.3	40.4 (21)
	non-Danish	3–20	15	14.5	58.5 (24)

### Legislation and welfare

All respondents were asked about any national legal requirements concerning housing, handling, and transportation of UO pigs. They were also asked if they considered some UO pigs less fit for transport and if some UO pigs need extra care or special treatment at the farm. Results are in Table 3.

**Table 3:** Questions regarding legal requirements, fitness for transport and housing

		Answer % (*n*)		
Question	Group	Don’t know	No	Yes
1) Are there any legal requirements in your country concerning housing, handling, and transportation of pigs with UO?	Danish	3.8 (2)	0	96.2 (50)
	Non-Danish	9.8 (4)	26.8 (11)	63.4 (26)
2) Do you think some pigs with UO are less fit for transport?	Danish	3.8 (2)	3.8 (2)	92.3 (48)
	Non-Danish	2.4 (1)	0	97.6 (40)
3) Do you think some pigs with UO need special treatment and extra care at the farm?	Danish	0	0	100 (52)
	Non-Danish	4.9 (2)	9.8 (4)	85.4 (35)

The 6 veterinarians answering “don’t know” in the question about legal requirements were all placed in European countries. Three were working in clinical practice and three were outside clinical practice (1 Danish, 2 non-Danish). Of the 11 veterinarians answering “no”, three were outside Europe, but the remaining eight were working in Europe (four within clinical practice).

**Fig. 3:**
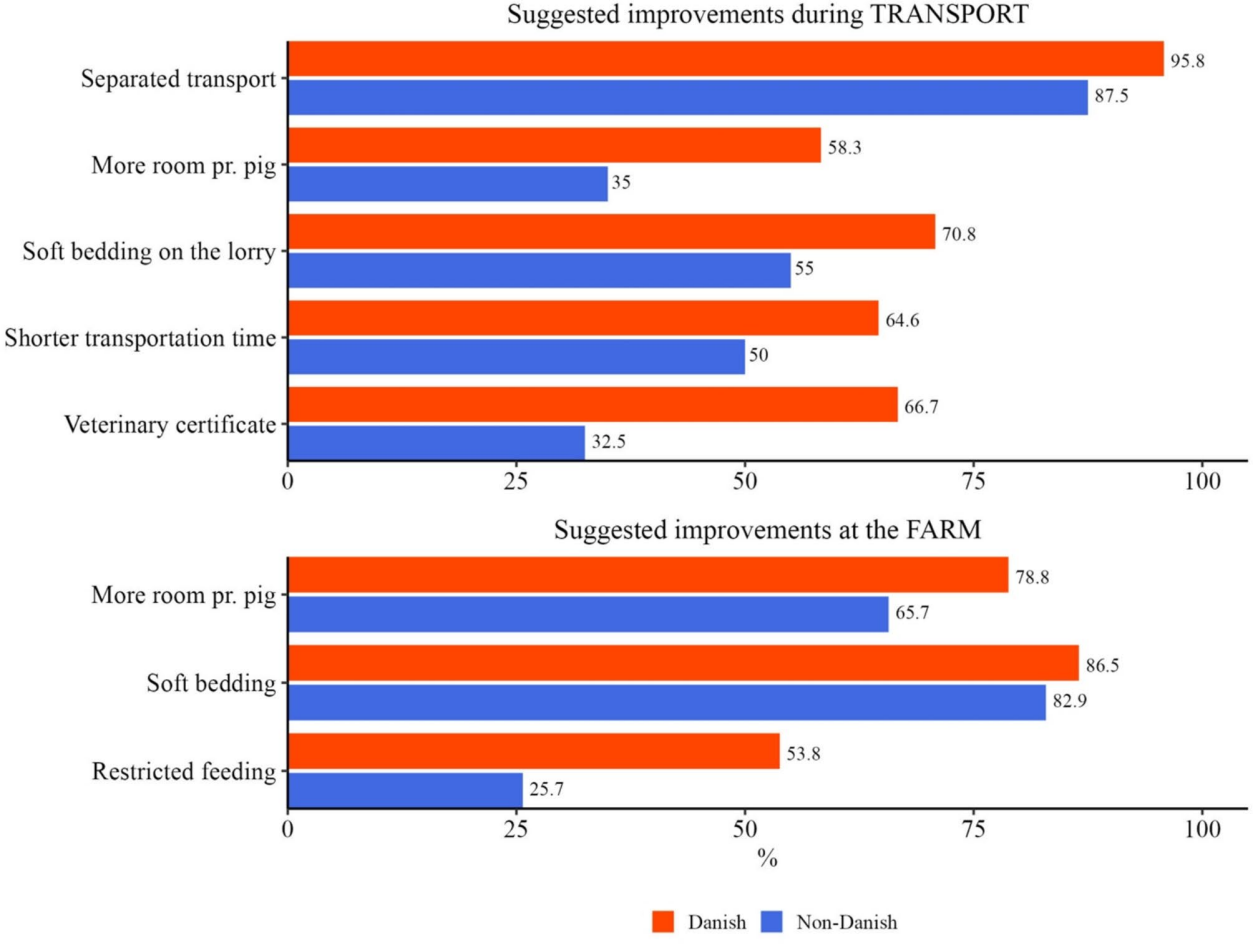
Suggested improvements during transport and at the farm; 88 (48 Danish, 40 non-Danish) veterinarians agreed that some UO pigs might be less fit for transport and 87 (52 Danish and 35 non-Danish) veterinarians agreed that some UO pigs might need special treatment

The veterinarians answering yes to questions 2 or 3 in Table 3, could tick several suggested improvements during transport/ at the farm. The results are in Fig. 3.

The veterinarians could also suggest improvements. One respondent asked for clear guidance on transport fitness and another thought that drivers should not be legally responsible for pigs with certificates. At the farm level, the following were suggested as extra care/ special treatment: Fiber-rich feed, deep bedding, clean/ dry environment, isolation/ sick pens, analgesia, good hygiene, surgery, and euthanasia.

### Significance of UO and risk factors for UO development

**Fig. 4:**
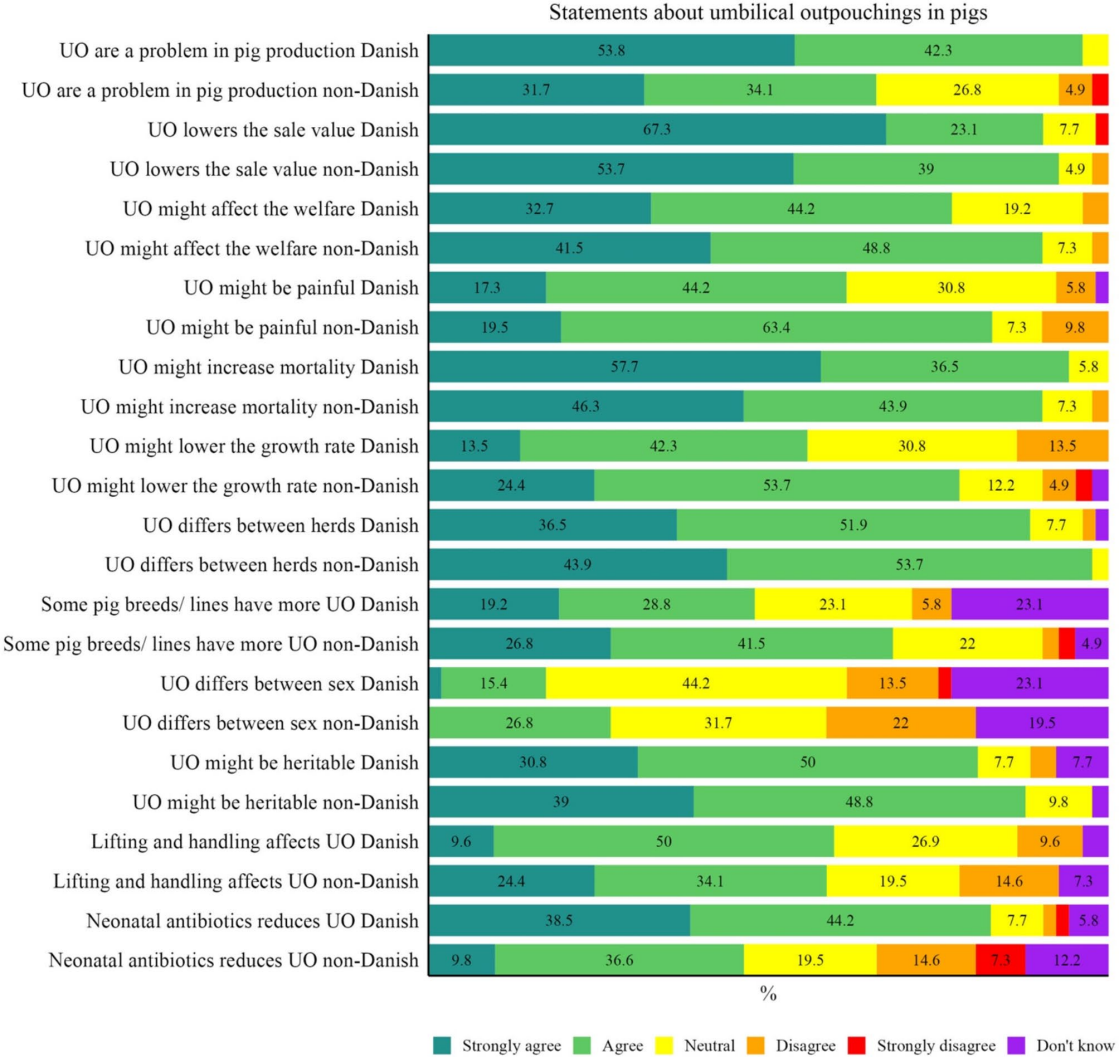
The veterinarians were asked about their opinion regarding a series of UO statements^1^

Figure 4 shows how strongly the veterinarians agreed to a series of questions regarding various aspects related to the significance of UOs and risk factors for UO development. Overall, the respondents either agreed or strongly agreed with the statements, except for the statement about whether UO differs between the sexes where they primarily disagreed.

Fifty-five veterinarians agreed or strongly agreed that some pig breeds/ lines had more UO. The Danish veterinarians mentioned Danbred, Danish Genetics, and Duroc, the non-Danish veterinarians also mentioned Danbred and Danish breeds, in addition to large white, duroc, alphagène, and fast-growing hybrids.

Twenty veterinarians (21.5%) stated that there were differences between male and female pigs, 11 (55%) stated male pigs have more problems, and 9 (45%) stated female pigs have more problems with UO.

A total of 55 (59.1%) veterinarians agreed that the way piglets are lifted and handled affects the risk of UO development. Their recommendations for lifting and handling piglets varied:

- Both hindlegs.
- Under the stomach.
- Under sternum.
- Not in hindlegs.

82.7% (77) of the veterinarians agreed that welfare could be affected and were requested to explain how UO affected animal welfare. Frequent answers were:

- Increased risk of intestinal disorder/ compromised intestinal function/ incarcerated intestines.
- Increased risk of aggression from other pigs/ injuries inflicted by pen mates.
- Affected ability to walk/ express normal behaviour.
- Risk of ulcers/ infection/ abscesses.
- Pain/ reduced general condition.

### Management of umbilical outpouchings

**Fig. 5:**
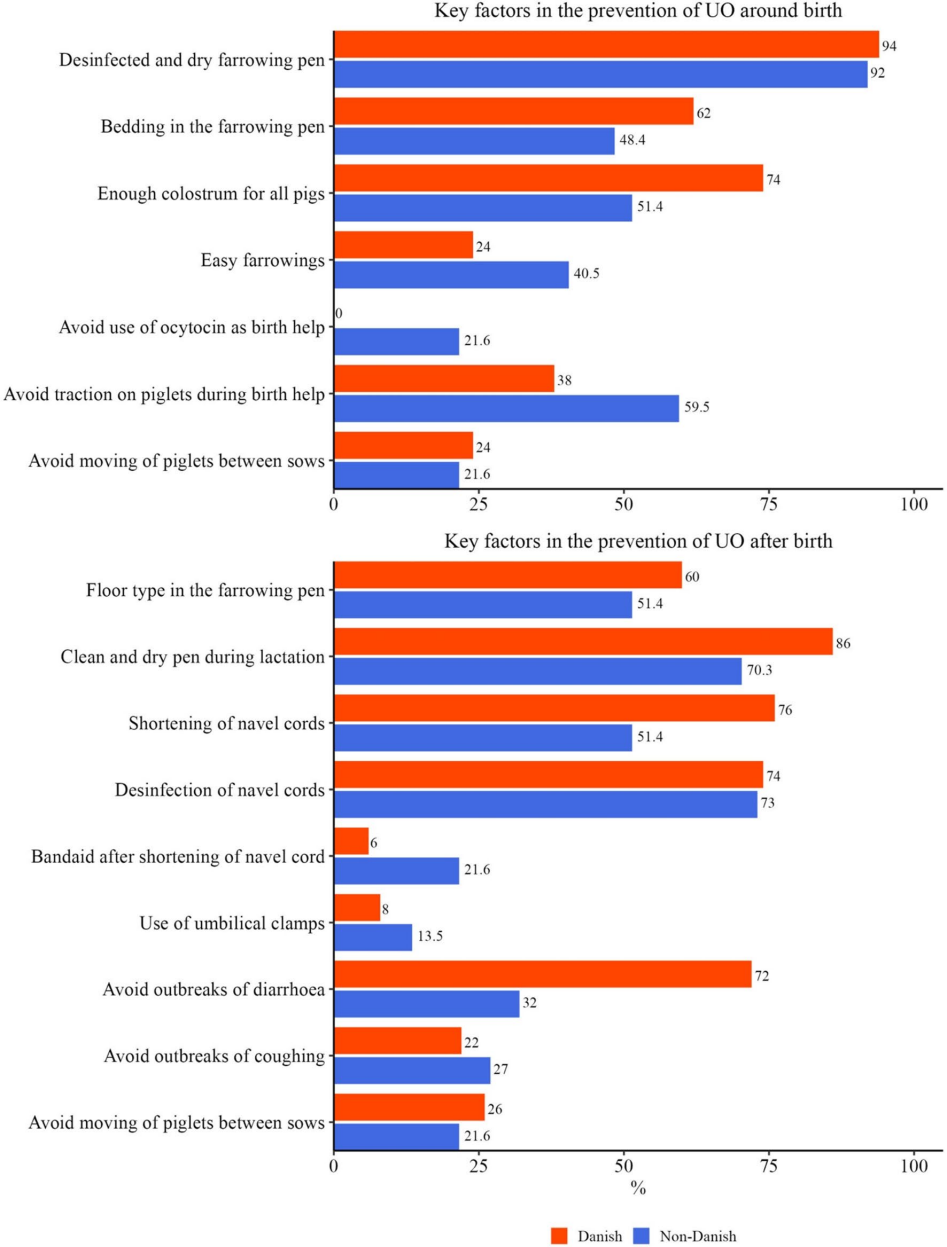
Key factors considered important by 50 Danish and 37 non-Danish veterinarians that would recommend preventive measures

The veterinarians were asked if they would recommend preventive measures against UO: Between Danish and non-Danish veterinarians 50 (96.2%) and 37 (90.2%) agreed to this. Figure 5 shows which statements those 87 veterinarians consider important in the prevention of UO before and around birth, as well as after birth. The veterinarians could also add alternatives, their suggestions included: Antibiotics on day 1, avoiding stress in piglets using gentle handling, no leg lifting, and avoiding pulling on the navel cord.

Bedding material was considered important by 49 (56.3%) veterinarians who stated powder-like materials as their preferred material (41.9% Danish, 33.3% non-Danish) and it was mentioned that it should be fine and soft and not inflict trauma or injury, secondly, it should keep the pen dry.

**Fig. 6:**
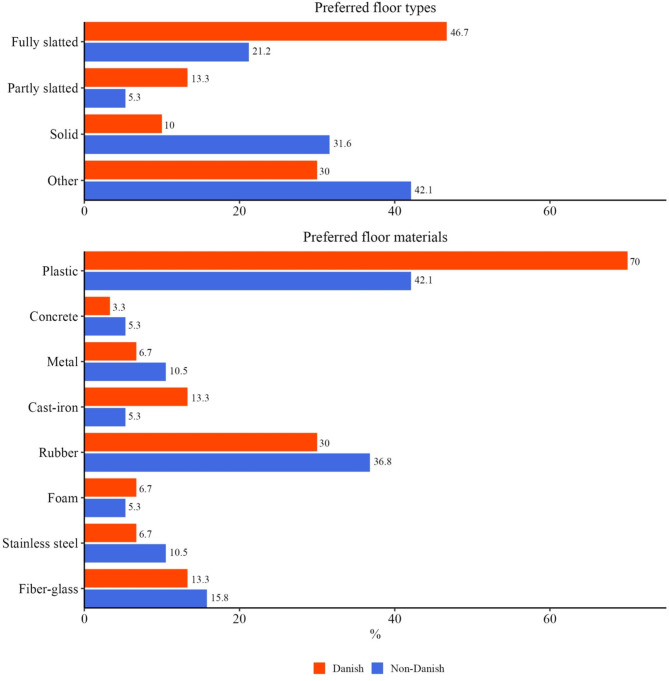
Floor types and floor materials preferred by 30 Danish and 19 non-Danish veterinarians

Floor type was also considered important by 49 veterinarians, that were asked what type of floor and floor material would reduce the occurrence of UO. The results are in Fig. 6. Under “other” the veterinarians specified that the floor needs to be clean and with a smooth surface.

**Fig. 7:**
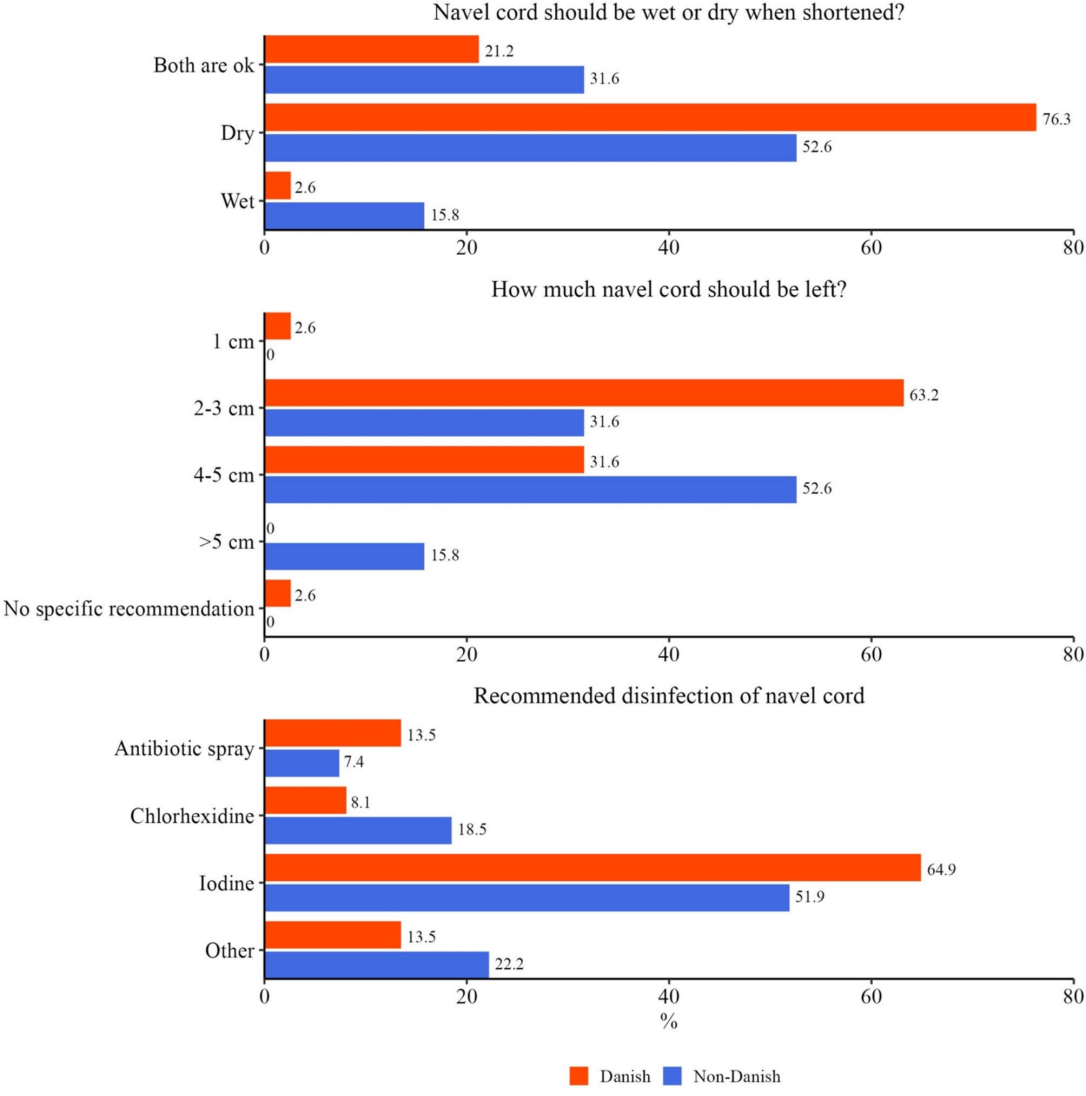
Recommendations for shortening (57 veterinarians) and disinfection of the navel cord (64 veterinarians)

Shortening of the navel cord was considered important by 38 (76%) Danish and 19 (51%) non-Danish veterinarians. Disinfection was considered important by 37 (74%) Danish and 27 (72%) non-Danish veterinarians. Results are shown in Fig. 7. Not all vets recommend a combination of shortening and disinfection.

Under antibiotic spray, chlortetracycline, oxytetracycline, and tetracycline were mentioned. Under other aluminium wound spray and wound (copper/ zinc) spray were mentioned.

**Fig. 8:**
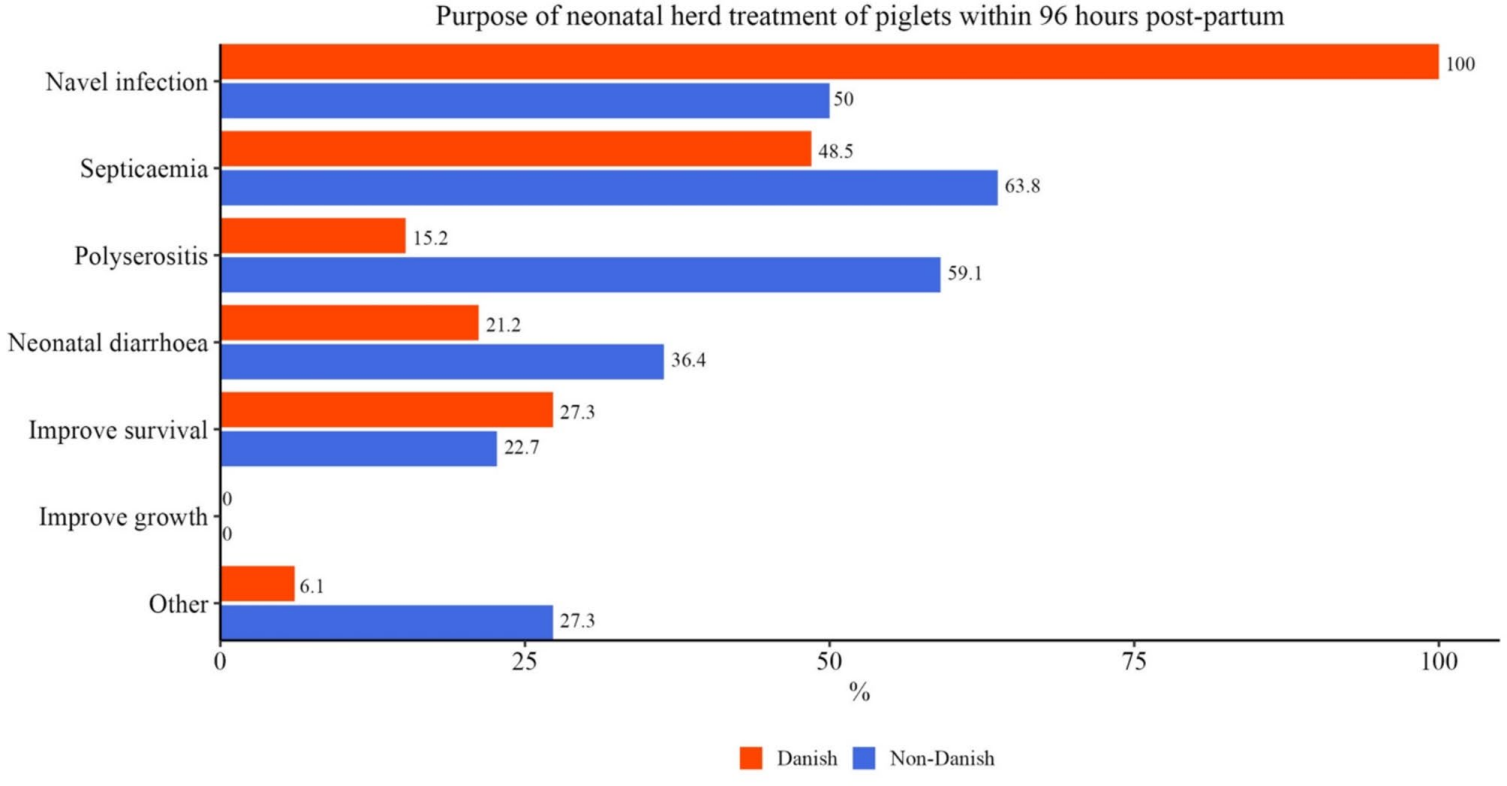
Purpose of neonatal herd treatment – 33 Danish veterinarians and 22 non-Danish veterinarians working in clinical practice

Only the 64 (34 Danish, 30 non-Danish) veterinarians working in clinical practice were asked the following questions: Do you prescribe antibiotics for herd treatments of piglets within the first 96 h? Secondly, they could choose several different indications, as well as make their own. Answers are in Table 4. Figure 8 shows the indications used for herd treatments of piglets. Other indications included: infections after tail docking, prevention of arthritis, reduction of morbidity/ mortality, and prevention/ control of *Streptococcus suis*/ *Actinobacillus pleuropneumoniae*.

**Table 4:** Prescription of antibiotics for herd treatment of piglets

	% (*n*)			
	No	Yes		
		100% of herds	25–99% of herds	< 25% of herds
Danish	2.9 (1)	20.6 (7)	73.5 (25)	2.9 (1)
Non-Danish	26.7 (8)	3.3 (1)	6.7 (2)	63.3 (19)

Danish veterinarians reported that herd treatment of all piglets was used once in the farrowing unit in 20–100% of the treated herds, twice in 5–65%, and more than twice in 5–20% of the herds.

The non-Danish veterinarians reported antibiotic usage once in 1–80% of the herds, twice in 3–30% and < 3% received more than two treatments.

The most prescribed substances in Denmark were amoxicillin (93.9%), tulathromycin (36.3%), and lincomycin/ spectinomycin (24.2%), and in non-Danish countries it was amoxicillin (63.6%), tulathromycin (40.9%), and penicillin (22.7%).

Lastly, veterinarians working in clinical practice were asked how they would treat a piglet/ weaner/ finisher with a large uncomplicated UO without ulcers.

**Fig. 9:**
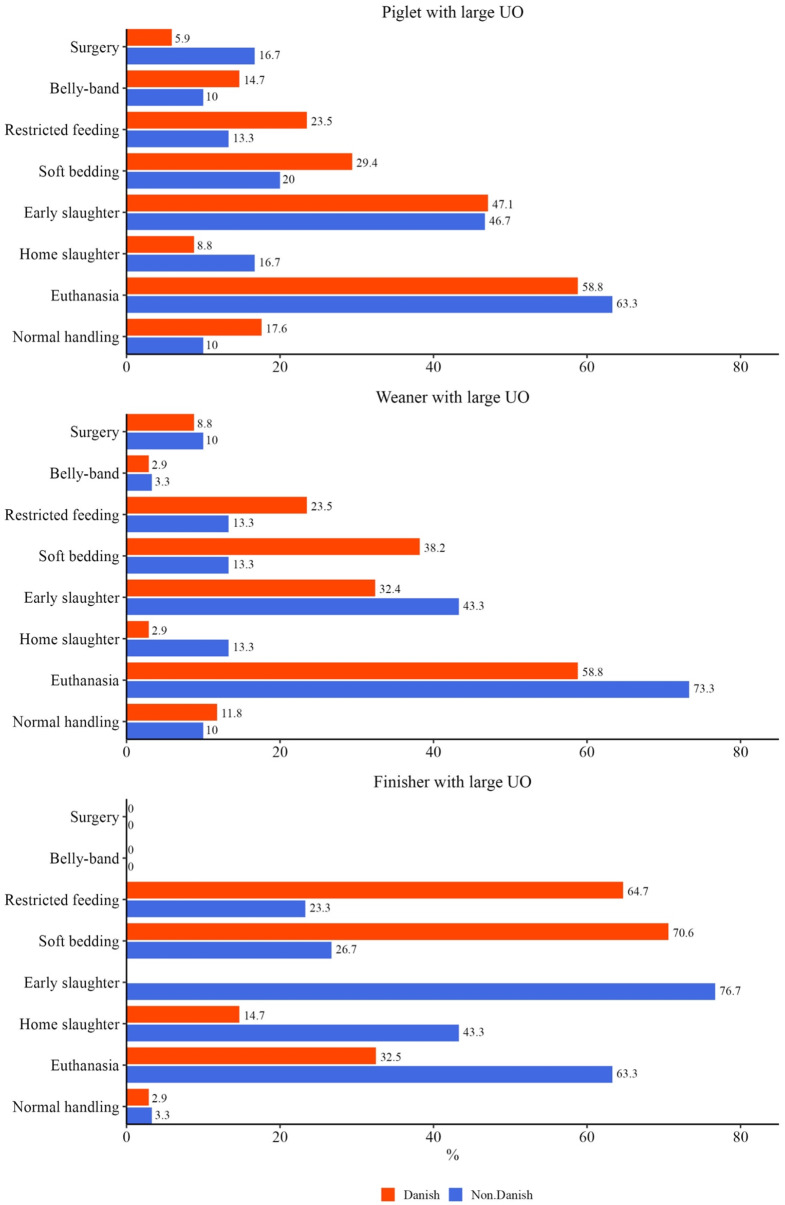
Answers from 64 veterinarians working in clinical practice: recommendations for the handling of piglet/ weaner/ finisher with large umbilical outpouchings

The answers are shown in Fig. 9. Among alternative solutions; “more room)” was mentioned under this.

## Discussion

A classical problem for all surveys is the fact that respondents reply for a reason, in this case, respondents are probably biased towards considering UOs in pigs a problem, which can explain the high agreement level in this survey. The number of respondents is also quite low considering we wished for replies from veterinarians working across all of Europe. We had access to email lists targeting Danish veterinarians working within porcine health management, but no such lists for the rest of the European Countries, therefore European veterinarians were targeted through ESPHM and EAPHM email lists. The low response from non-Danish veterinarians could indicate that UOs are not considered a problem in other countries.

The number of Danish respondents is equivalent to approximately a third of the Danish veterinarians working within porcine health management, those results are therefore likely reflective of Danish pig veterinarians. The same cannot be said for the non-Danish respondents, there are way too few for the results to be considered representative of the study population of European veterinarians, the results are however currently the only ones available.

### Fitness for transport

There were no significant differences between the fitness for transport evaluation made by Danish and non-Danish veterinarians. The only slight difference was found in one evaluation of a pig with an ulcer. The difference probably occurs because the Danish interpretation of the EU directive is a zero tolerance towards ulcers on the UO. This different interpretation of the effect of an ulcer on fitness for transport between Danish veterinarians and other nationalities is interesting and could show an objective for future research to objectively assess how ulcers affect or do not affect the fitness for transport.

When arranging the three statements relevant to transport fitness the size of the UO relative to the size of the pig was considered most important, followed by the UO affecting the ability to walk normally and the least important was how close the UO was to the ground.

When asked to specify a minimum distance from the UO to the floor the Danish and non-Danish had the same range and median for weaners, with a slightly larger mean from the non-Danish veterinarians. The results for finishers differed more with the non-Danish veterinarians having the largest range, median and mean. Having a specified size defined as problematic was supported by less than half of the Danish veterinarians and more than half of the non-Danish. The suggested size was centred around 10–15 cm.

### Legislation and welfare

Most of the Danish veterinarians were aware of legislation regarding UOs, whereas almost 40% of the non-Danish answered “don’t know” or “no”. This is somewhat surprising since nearly all respondents are working within Europe and as such should follow the rules stated in the EU directives [3, 4] and implemented in national legislation.

Almost all the veterinarians agreed that some UO pigs might need extra care during transport or in housing.

### Significance of UO and risk factors for UO development

Nearly all veterinarians agreed or strongly agreed with the series of statements mentioned in Fig. 4. Problems with UO seem to be affecting Danish veterinarians more than non-Danish. That might be caused by the Danish interpretation and implementation of the EU directives [3, 4].

Many veterinarians considered that UO might affect the welfare or might be painful for the pigs, especially the non-Danish veterinarians. There is very little literature investigating pain in pigs, especially UO pigs. However, one Danish study found altered behaviour in UO pigs during a stay in a pick-up facility, suggestive of experienced pain [14] and two more recent studies have found that many pigs with UO have complications to their UO, such as adhesions, incarcerations, and bleedings [15, 16].

Increased mortality is also caused by UO, maybe not directly, but at least indirectly as many veterinarians recommend euthanasia of UO pigs. Reduced growth rate as well as increased mortality of UO pigs is described [17].

Differences between herds have also been documented [1], however, whether differences are biological or more related to management is still uncovered.

Differences between pig breeds/ lines are not fully investigated but there are indications that genetics play a role [12, 18, 19]. Only a minority of the respondents agreed that sex plays a role in UO development, despite newer research showing that female pigs are at higher risk [9, 20, 21].

There is currently no peer-reviewed research investigating how handling and lifting piglets affect UO development. The effects of neonatal antibiotic treatment on UO development are showing mixed results, some find an effect [11], but most have no effect [9, 10, 12, 13].

### Management of UO

Almost all veterinarians would recommend preventive measures against UO and the recommendations between Danish and non-Danish veterinarians are similar, however slight differences were seen in the recommendations around farrowing where the non-Danish veterinarians mark easy farrowing, no oxytocin for sows, and avoiding traction of piglets during birth help.

The results for floor type were mixed, but the overall conclusion was that the floor needs to be clean. For floor materials, it seems that there was a preference towards softer floors (plastic and rubber). Recommendations for shortening and disinfection of the navel cord were high, despite no peer-reviewed studies in pigs showing the effect of either; iodine was the preferred substance for disinfection.

The use of neonatal antibiotics was considerably higher among Danish veterinarians compared to non-Danish. The indications for treatments differed as well with prevention of navel infections being twice as high among Danish veterinarians compared to non-Danish. The most prescribed substances were amoxicillin and tulathromycin in both groups.

The recommendations for the treatment of UO pigs in stable differed between the three age groups. Surgery was only recommended for piglets and weaners. Restricted feeding and bedding seemed to be mainly used in Denmark. Early slaughter and home slaughter seemed to be more recommended by non-Danish veterinarians, maybe because home slaughtering in Denmark is only allowed for private use and reselling is prohibited [22]. Euthanasia is recommended more by non-Danish veterinarians, than by Danish.

## Conclusions

Umbilical outpouchings are perceived as a challenge in pork production; affecting the welfare of the individual pig as well as the management of the entire production. There were no significant differences between the veterinarians’ assessments of fitness for transport between Danish and non-Danish veterinarians, and nearly all the veterinarians agreed that some UO pigs might need special attention and care. Most veterinarians would recommend preventive measures. Despite most respondents in this survey working under the laws of the European Union, some were unaware of the legislation regarding UO pigs.

### Methods

#### Study Design

SurveyXact was used to conduct a questionnaire survey among veterinarians. The survey was launched in June 2021 and closed on January 12th, 2022. The studied population were swine veterinarians either members of the European College of Porcine Health Management (ECPHM), European Association of Porcine Health Management (EAPHM) or Danish veterinarians. In June 2021 the newsletter of ECPHM included a short introduction to the survey and a link to the questionnaire (number of recipients unknown), in July the same applied to the newsletter of EAPHM (596 recipients) and on August 30th, 2021, the link was sent in a direct email from EAPHM.

E-mails were sent to the SEGES^2^ list of Danish swine veterinarians on June 22nd (250 recipients) and repeated in August 2022. On September 6th the link was shared in “Faggruppe Svin”^3^ on Facebook (203 members) and “DVHS”^4^ Facebook shared the link on September 8th (302 members). The last sharing of the link was on September 8th in a “What’s App group” for ECPHM residents (38 members). All use of email lists was executed by mentioned organizations and the authors had no access to the email lists. Many recipients are probably the same across the different lists (e.g. ECPHM and EAPHMs lists, Danish Facebook groups and SEGES list), the total number of unique recipients of the survey is thus unknown.

The reporting of results was divided into Danish and non-Danish respondents to account for the dominance of Danish respondents.

### Questionnaire

The questionnaire was designed according to DH Stone’s directions [23]. It consisted of a combination of closed and open questions and statements to which the veterinarians either agreed or disagreed on a Likert scale with different levels dependent upon the question. The questionnaire was organised into subjects on fitness for transport, welfare, and management of UO. If the participants answered affirmatively to the questions, they were asked additional questions; if they answered no, they were not asked further. E.g. the series of statements would trigger further questions dependent upon the respondents’ answers.

### Statistical analysis

Only complete responses are reported in this study. All data were visualized, and graphs were made, in Rstudio [24]. Differences between groups were examined for significance using 2 × 2 tables and p values below 0.05 were considered significant.

## Data Availability

No datasets were generated or analysed during the current study.

## Footnotes

1 Statements are shortened in the Figure compared to the survey; the meaning is the same

2 Danish Pig Research Centre, Danish Agriculture and Food Council.

3 Danish Veterinary Association Profession Pig.

4 Dansk Veterinær Hyologisk Selskab/ Danish Pig Veterinary Society.
